# Large screening of CA-MRSA among *Staphylococcus aureus *colonizing healthy young children living in two areas (urban and rural) of Portugal

**DOI:** 10.1186/1471-2334-10-110

**Published:** 2010-05-03

**Authors:** Débora A Tavares, Raquel Sá-Leão, Maria Miragaia, Hermínia de Lencastre

**Affiliations:** 1 Laboratory of Molecular Genetics, Instituto de Tecnologia Química e Biológica (ITQB), Universidade Nova de Lisboa (UNL), Oeiras, Portugal; 2 Centro de Matemática e Aplicações Fundamentais (CMAF), Universidade de Lisboa, Lisboa, Portugal; 3 Laboratory of Microbiology, The Rockefeller University, New York, NY, USA

## Abstract

**Background:**

The incidence of pediatric infections due to community-associated methicillin-resistant *Staphylococcus aureus *(CA-MRSA), including children with no identifiable risk factors, has increased worldwide in the last decade. This suggests that healthy children may constitute a reservoir of MRSA in the community. In this study, nested within a larger one on nasopharyngeal ecology, we aimed to: (i) evaluate the prevalence of MRSA colonizing young children in Portugal; and (ii) compare results with those obtained in a study conducted a decade ago, when this prevalence was <0.5%.

**Methods:**

In the years 2006, 2007, and 2009, nasopharyngeal samples were obtained from 2,100 children aged up to 6 years attending day-care centers. *S. aureus *were isolated by routine procedures and strains were tested for susceptibility against a panel of 12 antimicrobial agents. MRSA isolates were further characterized by SmaI-PFGE profiling, MLST, *spa *typing, SCC*mec *typing, and presence of virulence factors.

**Results:**

Seventeen percent of the children carried *S. aureus*. Among the 365 isolates, non-susceptibility rates were 88% to penicillin, 14% to erythromycin, 6% to clindamycin, 2% to tetracycline, and <1% to oxacillin, rifampicin, ciprofloxacin, and SXT. Three MRSA strains were isolated. These had properties of CA-MRSA, such as low-level resistance to oxacillin and limited resistance to non-beta-lactams. Two CA-MRSA were related to USA700 (ST72-IV): one was ST72-IVc, *spa *type t148; the other was ST939-IVa (ST939 is a single locus variant (SLV) of ST72), *spa *type t324. The third strain was related to USA300 (ST8-IV) being characterized by ST931 (SLV of ST8)-VI, *spa *type t008. The three MRSA strains were PVL-negative, but all carried LukE-LukD leukocidin, hemolysins gamma, gamma variant and beta, and staphylococcal enterotoxin *sel*.

**Conclusions:**

Our results, based on analysis of *S. aureus *isolated from nasopharyngeal samples, suggest that in Portugal the prevalence of CA-MRSA carriage in healthy young children remains extremely low favoring the exclusion of this group as a reservoir of such isolates.

## Background

*Staphylococcus aureus *colonizes asymptomatically mainly the anterior nares of humans. It is also a frequent cause of clinically important infections [1]. Methicillin-resistant *S. aureus *(MRSA) emerged in the 1960s, shortly after the introduction of methicillin in clinical practice, through the acquisition of the *mecA *gene. The gene *mecA *encodes for a low affinity penicillin binding protein, PBP2A, and is carried by the staphylococcal chromosomal cassette *mec *(SCC*mec*) [2,3]. Molecular characterization of a large collection of isolates from different geographic regions showed that SCC*mec *has been acquired in more than one event by already epidemic genetic backgrounds, giving rise to five main pandemic clones that have spread worldwide [4,5]. Nowadays, MRSA prevalence in hospitals can range from less than 5% in countries from Northern Europe and The Netherlands, to more than 70% in Japan and Hong Kong [6,7].

Three decades after the emergence of MRSA, when infection control policies aimed to decrease the prevalence of MRSA in hospitals of several countries were being implemented MRSA changed its epidemiology. From a restricted hospital-associated pathogen, MRSA made its way into the community [8]. During the last decade, an increasing incidence of infections in the community due to MRSA has been reported [9]. In particular, pediatric infections due to community-associated MRSA (CA-MRSA) among children with no identifiable predisposing risks for health-care associated infection have been documented worldwide [10-12]. A major and unsettled issue in the worldwide emergence of CA-MRSA is the source of such isolates in the community. The above-mentioned observations led some authors to propose that healthy children might constitute an asymptomatic reservoir of CA-MRSA [13].

While numerous surveillance programs have been producing information on resistance patterns and molecular types of *S. aureus *in hospitals, relatively little information is available on strains of this species that colonize healthy human populations. Information on this issue has become particularly important with the worldwide emergence of drug resistant strains - particularly epidemic MRSA lineages - outside hospitals, in the "healthy" human community.

In Portugal, the prevalence of nosocomial MRSA varies widely between hospitals but, overall, remains among the highest (53%) in Europe [6,14]. Moreover, the prevalence of MRSA causing skin and soft tissue infections (SSTIs) in Portugal was the highest in Europe between 1998 and 2004 [15]. However, the carriage and infection rates of MRSA in the community remain mostly unknown.

An earlier study performed in Portugal on the composition of *S. aureus *flora colonizing healthy human populations provided a striking demonstration on the virtual absence of MRSA in healthy humans in the late 1990-s [16], i.e., at a time when MRSA strains were already contributing to over 50% of *S. aureus *infections in Portuguese hospitals. Indeed, of five MRSA out of 285 *S. aureus *isolates recovered from 2,111 children in that study, three were related to nosocomial clones. The other two were of ST82 and had low-level resistance to oxacillin [16].

In this report we examined a large collection of nasopharyngeal samples from healthy children taken between 2006 and 2009 for *S. aureus *and for the presence and/or frequency of MRSA strains. The comparison of this time period with the previous study encompasses the worldwide emergence of CA-MRSA and thus should provide an unbiased sample to monitor whether significant changes in the representation of MRSA in this population have occurred. To achieve such goal, we enrolled over 2,000 children between 2006 and 2009. Participants were enrolled in two areas: Oeiras (urban municipality) and Montemor-o-Novo (rural municipality). To our best knowledge, this is one of the largest studies conducted so far worldwide aiming to identify CA-MRSA in this risk group.

## Methods

In the winter months of January-March of 2006, 2007, and 2009, as part of an ongoing study on colonization patterns by *Streptococcus pneumoniae *(R. Sá-Leão, unpublished data, [17] nasopharyngeal swabs were collected from children aged 4 months to 6 years attending DCCs in the urban area of Oeiras. In 2009, the study was extended to the rural area of Montemor-o-Novo. The DCCs were selected to cover the spectrum of social backgrounds found in both areas. Approval for the study was obtained from the Ministry of Education and Directors of DCCs. Parents or guardians gave written informed consent, and a questionnaire was filled in with information on illnesses, antibiotic consumption, and attendance to hospitals within the six months preceding sampling.

In each year, a single sample was collected from each child. Overall, 2,100 samples were obtained: 571 in 2006, 538 in 2007, and 991 in 2009 (611 from Oeiras and 380 from Montemor-o-Novo).

Nasopharyngeal swabs were taken as described [17], and inoculated within four hours onto mannitol salt agar (Difco, Detroit, MI). Plates were incubated in aerobic conditions for 18 h-48 h at 37°C. Single morphologically distinct mannitol-positive colonies were isolated and pure cultures were tested for coagulase production by using Staphytect Plus (Oxoid, Hampshire, England), according to the manufacturers' instructions. Cultures that were mannitol-positive and coagulase-negative were also tested with Coagulase Plasma Rabbit (Becton, Dickinson and Company, Sparks, MD), according to the manufacturers' instructions. Cultures that were mannitol-positive and coagulase-positive were considered to be *S. aureus*.

Antimicrobial susceptibility to penicillin, oxacillin, erythromycin, clindamycin, tetracycline, vancomycin, ciprofloxacin, rifampicin, linezolid, quinupristin/dalfopristin, gentamicin, and trimethropim/sulphamethoxazole (SXT) was tested by disk diffusion, according to the Clinical Laboratory Standards Institute recommendations and definitions for all *S. aureus *isolates[18]. Disks were purchased from Oxoid, Hampshire, England. Isolates were classified as either susceptible or non-susceptible, the latter classification including isolates with both intermediate and resistant phenotypes.

All isolates showing an inhibition zone smaller than 20 mm surrounding the oxacillin disk were screened by PCR for the presence of the *mecA *gene using primers mecAP4 and mecAP7 [19]. MICs to oxacillin were determined for all isolates and to vancomycin for MRSA isolates only; MICs were determined by Etest (AB Biodisk, Slona, Sweden).

MRSA isolates were further characterized by pulsed-field gel electrophoresis (PFGE) [20], *spa *typing [21], multilocus sequence typing (MLST) [22], and SCC*mec *typing [4,23-25]. The presence of genes coding for virulence factors Panton-Valentine leukocidin (*pvl*), LukE-LukD leukocidin (*lukED*), class F leukocidin (*lukM*), staphylococcal enterotoxins (*sea*-*e*, *seg*-*j*, *sep*, *sel*), toxic shock syndrome toxin (*tsst*), exfoliative toxins (*eta*, *etb*, *etd*), and hemolysins (gamma [*hlg*], gamma variant [*hlgv*], and beta [*hlb*]) was screened by multiplex PCR reactions as previously described [26-28].

The two MRSA isolates recovered from children in 1997 (during the previous study) that were not associated with nosocomial clones were also characterized by *spa *typing and SCC*mec *typing, and the presence for virulence genes was screened, as above [16].

## Results

### *S. aureus *carriage

A total of 2,100 samples were obtained. The mean age of the participants was 3.5 years and 52.4% were male. *S. aureus *were isolated from 17.4% of the 2,100 samples, ranging from 13.2% to 21.6% depending on the sampling year and origin of isolates (Table 1). In line with previous observations, [29-31] carriage of *S. aureus *was significantly associated with age ranging from 6.3% among children aged less than two years and steadily increasing up to 27.5% among those aged six years (data not shown).

**Table 1:** Study collection and distribution of *S. aureus *isolates

Year	Geographic origin	Day care centers	No. (%)		
			
			Nasopharyngeal samples	*S. aureus*	MRSA
2006	Oeiras	11	571	92 (16.1)	3 (0.53)
2007	Oeiras	11	538	71 (13.2)	0
2009	Oeiras	9	611	120 (19.6)	0
2009	Montemor-o-Novo	16	380	82 (21.6)	0
		
		Total	2,100	365 (17.4)	3 (0.14)

### Antimicrobial susceptibility

Among the 365 *S. aureus*, 11.5% were susceptible to all antibiotics tested and the frequency did not vary significantly within the four collections.

Non-susceptibility rates were 88.5% to penicillin, 14.0% to erythromycin, 5.5% to clindamycin, 2.2% to tetracycline, and <1% to oxacillin (three isolates), rifampicin (three isolates), ciprofloxacin (two isolates), and SXT (single isolate). All isolates were susceptible to vancomycin, linezolid, quinupristin/dalfopristin, and gentamicin. Antibiotic resistant rates to individual antibiotics were not significantly different within the four collections.

### MRSA carriage

The three oxacillin-resistant isolates were confirmed to carry the *mecA *gene and to have low-level resistance to this antibiotic (Table 2). The MRSA isolates were recovered from girls (two aged 3, and one aged 5 years) attending different DCCs in Oeiras in 2006. One girl had received three or more courses of antibiotics in the six months preceding sampling. None had attended a hospital in the previous six months. Other risk factors associated with carriage of CA-MRSA were not investigated.

**Table 2:** Characterization of MRSA strains

Strain ID	Isolation year	Antibiotype^a^ (resistant to)	Oxacillin MIC (mg/L)	MLST	PFGE	*spa *type		SCC *mec *type	Virulence factors^b^	Representative of
									
						RIDOM	eGenomics			
DCC5292	2006	PEN, OXA	24	939	A_1_	t324	451 (UJGGMDMGGM)	IVa	*lukED, hlg, hlgv, hlb, sel*	USA700
DCC5504	2006	PEN, OXA	2	931	B	t008	1049 (YHGFMBOBLO)	VI	*lukED, hlg, hlgv, hlb, sel*	USA300
DCC5723	2006	PEN, OXA, ERY, CLI	8	72	A_2_	t148	193 (UJGFGMDMGGM)	IVc	*lukED, hlg, hlgv, hlb, sel*	USA700
DCC1028	1997	PEN, OXA, ERY	12	82	C_1_	t186	9 (UGFMEEBBPB)	IVa	*lukED, hlgv, hlb, sel, eta*	
DCC1076	1997	PEN, OXA, ERY, GEN	24	82	C_2_	t3221	1190 (UGEBBPB)	IVc	*lukED, sep, hlg, hlgv, sel*	

^a^PEN, penicillin; OXA, oxacillin; ERY, erythromycin; CLI, clindamycin; GEN, gentamicin

^b^Virulence factors tested: Panton-Valentine leukocidin (*pvl*), LukE-LukD leukocidin (*lukED*), class F leukocidin (*lukM*), staphylococcal enterotoxins (*sea-e, seg-j, sel, sep*), toxic shock syndrome toxin (*tsst*), exfoliative toxins (*eta, etb, etd*), and hemolysins (gamma [*hlg*], gamma variant [*hlgv*], and beta [*hlb*])

### Molecular characterization of MRSA

**Figure 1:**
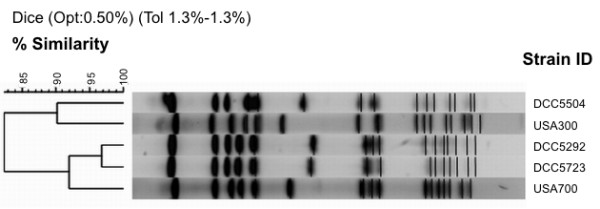
**PFGE pattern of MRSA isolates**. Dendrogram resulting from the comparison of PFGE band restriction patterns of the three MRSA strains with the band patterns of prototype strains of USA300 and USA700 clones. The dendrogram was generated by the BioNumerics software (version 5.10, Applied Maths, Gent, Belgium). Patterns were clustered by UPGMA using the Dice similarity coefficient with an optimization of 0.50% and a tolerance of 1.3%. PFGE types (clusters) were defined based on a similarity of 80% or higher.

Molecular characterization was done for the three MRSA isolates identified in this study as well as for the two ST82 identified in the 1996-1998 study [16]. Two MRSA strains isolated in 2006 were, by PFGE, related to USA700 (ST72-IV): one strain was of ST939-IVa, *spa *type t324; the other was of ST72-IVc, *spa *type t148 (Figure 1, Table 2). ST939 is a single-locus variant (SLV) of ST72. The *spa *types are single repeat variants among each other and both differ in three repeats from the *spa *type of USA700 prototype strain (t126).

The third MRSA strain isolated in 2006 was, by PFGE, related to USA300 (ST8-IV). This isolate was of ST931-VI, *spa *type t008. ST931 is a SLV of ST8 and *spa *type t008 has been associated with USA300 representatives. Of notice, this strain had a MIC to oxacillin of 2 mg/L, below the indicative breakpoint of 4 mg/L.

The three MRSA strains were PVL-negative, but all carried five other virulence genes: *lukED*, *hlg*, *hlgv*, *hlb*, and *sel *(Table 2).

The characterization of the two ST82 isolates described in the 1996-1998 study confirmed they were related to each other but not identical: strain DCC1028 was of *spa *type t186 and carried SCC*mec*IVa and the genes *lukED*, *hlgv*, *hlb*, *sel*, and *eta*; strain DCC1076 was of *spa *type t3221 and carried SCC*mec*IVc and the genes *lukED*, *hlg*, *hlb*, *sel*, and *sep*. Both were PVL-negative. The *spa *types differed among each other in an insertion/deletion of three repeats (Table 2).

## Discussion

During the last ten years we have been following the impact of antibiotic use and the introduction of the pneumococcal conjugate vaccine on the bacterial flora colonizing the nasopharynx of preschool children [17,32]. Although the nasopharyngeal sampling in these studies was designed to recover primarily the major microbial colonizers of the nasopharyngeal space, such as *Streptococcus pneumoniae*, *Haemophilus influenzae*, and *Moraxella catarrhalis*, the method also picks up a sample of other natural inhabitants of the upper respiratory tract - such as *S. aureus*. Thus, nasopharyngeal samples recovered from children in our surveillance program could also serve as a valuable source of information on possible changes that may have occurred in the composition of the natural flora of important human opportunistic pathogens, namely *S. aureus*.

Previous reports of CA-MRSA infections among children with no health-care associated risk factors provided evidence of a hospital-independent community reservoir [10-12,33]. In addition, other recent studies have identified risk factors for carriage and transmission of CA-MRSA, and, consequently, populations at higher risk of CA-MRSA infections due to their life styles. Due to crowded conditions, intensive exposure to antimicrobial agents, and close contact, DCC attendees have also been considered a high-risk population for carriage of MRSA [8]. Combined with an increasing incidence of infections in children with no predisposing risks factors, this population has been pointed as a possible community reservoir of CA-MRSA. In fact, recent studies from Greece and Japan reported rates of MRSA nasal carriage of 3.3% and 3.7%, respectively, corresponding to 5.5% and 9.1% of MRSA among total *S. aureus *isolates [13,34]. In Nashville, TN, and Taiwan MRSA nasal carriage rates have reached 7% to 9% of the sampled population (corresponding to 25-30% of MRSA among all *S. aureus *isolates) [29,35]. Finally, studies from Brazil and Illinois, IL, reported rates of MRSA nasopharyngeal carriage of 1.0% and 1.7%, respectively, corresponding to 7.5% and 9.3% of MRSA among total *S. aureus *isolates [36,37].

In the current study conducted among healthy children living in the Oeiras and Montemor-o-Novo municipalities of Portugal, we sampled a large group of children attending DCCs. As in the first study, nasopharyngeal MRSA colonization was found to be very low (0.24% in 1996-1998 and 0.13% in 2006-2009) [16]. Although we sampled the nasopharynx, which is not the preferred niche for *S. aureus *colonization, the proportion of MRSA among total *S. aureus *isolates was still very low: 1.77% in 1996-1998 and 0.82% in 2006-2009. This was a surprisingly low rate, in light of findings from other countries and knowledge of high prevalence of HA-MRSA in Portugal. However, one of the geographical areas studied is an urban area densely populated where use of antibiotics is intense and transmission of potential bacterial pathogens among children is known to be high and has been well documented [17]. In addition, in contrast to other European countries, in Portugal over 75% of pre-school children are attending DCCs [17], often since the early age of 4-5 months, five days a week, eight hours a day. Thus, our results may well represent the Portuguese reality.

Another recent study by Lamaro-Cardoso and co-workers also found a low prevalence (1.2%) of MRSA among DCC attendees in Brazil. Excluding the fact that the annual median household income of this population was below the Brazilian poverty line, only 4 of 14 MRSA isolates were not associated to health-care settings, and all had been recovered from children with at least one risk factor [38].

The three MRSA isolates recovered in the present study had properties of typical CA-MRSA such as low-level resistance to oxacillin and limited resistance to non-β-lactams [9]. Furthermore, by molecular typing they were identified as related to CA-MRSA clones USA300, a major epidemic CA-MRSA clone in the USA that is also spreading in Europe and USA700, a clone that has been associated to community and health-care settings [39,40]. The MRSA strain related to USA300 carried SCC*mec *type VI, which was first described in a nosocomial MRSA clone identified in Portugal [41]. The two ST82 strains found a decade ago also showed low-level resistance to oxacillin and limited resistance to non-β-lactams. In addition, they carried SCC*mec *IV, also characteristic of CA-MRSA. Of interest, the strains isolated in 2006 were unrelated to these ones. However, MRSA strains of ST88-IV (ST88 is a SLV of ST82) have been isolated in Portugal and China in the hospital setting, and in Nigeria and Switzerland in the community [42-46]. The emergence of a USA300 related clone among healthy children attending DCC is worrisome given its acknowledged high capacity of dissemination that can be rapidly amplified by the high rates of child-to-child transmission occurring in DCCs.

This study has some limitations. First, we sampled the nasopharynx, which is not the preferential niche of *S. aureus *and thus we probably underestimated the true prevalence of MRSA. In particular, we did not sample other sites of colonization such as anterior nares, throat, groin, and rectum [47]. Secondly, the study included only children aged up to six years who are less prone to be colonized by *S. aureus *than older children. Thirdly, we focused mainly in one urban region, even though a rural region was also studied in 2009. Although similar results were found over time and in both geographic locations, it remains unknown whether the observations made are representative of Portugal. Regardless of these limitations, we consider that this study contains valuable information on the evolution of MRSA carriage in asymptomatic children.

## Conclusions

Our results, based on the analysis of *S. aureus *isolated from nasopharyngeal samples, suggest that in Portugal the prevalence of CA-MRSA carriage in healthy young children remains extremely low favoring the exclusion of this group as a reservoir of such isolates. Given the current concern about CA-MRSA, the observed emergence of a USA300 related clone in this study, and the high prevalence of MRSA causing SSTIs in Portugal [15], an extension of these studies to other risk groups and continuous surveillance is mandatory.

## Competing interests

The authors declare that they have no competing interests.

## Authors' contributions

DAT performed experimental work and drafted the manuscript. RSL, MM, and HML designed the study. RSL helped to draft the manuscript. MM and HML critically revised the manuscript. All authors have read and approved the final manuscript.

## Pre-publication history

The pre-publication history for this paper can be accessed here:

http://www.biomedcentral.com/1471-2334/10/110/prepub
